# Assessing the Effect of Infant Peanut Exposure Guidelines on Peanut Aspiration Using Google Trends Search Analysis

**DOI:** 10.7759/cureus.68118

**Published:** 2024-08-29

**Authors:** Dana N Eitan, Bryant O Noss, Shauna R Schroeder, Patrick Scheffler

**Affiliations:** 1 School of Medicine, Creighton University School of Medicine, Phoenix, USA; 2 Department of Gastroenterology, Phoenix Children’s Hospital, Phoenix, USA; 3 Department of Otolaryngology, Head and Neck Surgery, Phoenix Children’s Hospital, Phoenix, USA

**Keywords:** allergy prevention in children, pediatrics, aspiration risk, google trend, peanut allergy

## Abstract

Background

The American Academy of Pediatrics (AAP) changed guidelines in 2017 to recommend introducing infants to peanuts as early as four months. Peanuts are also one of the most commonly aspirated food-related foreign bodies. Google Trends search pattern analysis has been validated in epidemiologic research as being reflective of healthcare events.

Methodology

Google Trends measures the popularity of a search term in a given week compared to the popularity of all search terms in that week, calculated as relative search volume (RSV), yielding a value between 0 and 100. We compiled peanut aspiration-related search data from January 2012 to January 2022 and compared the relative popularity of these searches before the change in guidelines to after.

Results

All queried search terms significantly increased in RSV when comparing the five years before and following January 2017. When pooling all terms, the median RSV increase was 13.8% (p < 0.001). “Choke on peanut” and the combination of peanut and cough had the highest median RSV increases from 9.6 to 17 and 50.8 to 63.1, respectively.

Conclusions

The change in 2017 of the AAP guidelines on early childhood peanut exposure was associated with an increase in online searches for peanut aspiration. This may be reflective of increased aspiration events, or possibly increased concern for aspiration. Current results support the need to closely counsel families on infant-safe peanut products to prevent dangerous aspiration events.

## Introduction

Peanuts have been found to be the most commonly aspirated organic foreign body in children, accounting for approximately 36-55% of all foreign bodies in children [1-3]. Young children are more likely to aspirate hard and small foods such as peanuts due to incomplete development of molar teeth as well as inadequacy of airway-protective mechanisms during swallowing [1,3,4]. In February 2017, the American Academy of Pediatrics (AAP) changed guidelines to recommend the early introduction of peanuts to infants as early as four months to reduce the risk of peanut allergies [5,6]. This change was based on the results of the Learning Early About Peanut Allergy (LEAP) trial, discovering an 86.1% relative reduction in peanut allergy prevalence in children with an initial negative skin prick test and a 70% relative reduction in children with a positive skin test [5].

Google Trends monitors public interest by analyzing specific search terms that were most popular in a given period. Data related to public use of the internet for health-related information has been increasingly utilized in scientific research and is considered to be a reliable insight into public attention since “72% of United States adults reported seeking health information online over the course of a year” [7,8]. For example, an epidemiological study by Seifter et al. determined that search terms such as “tick bite” and “cough” were deemed sufficient indicators of Lyme disease activity peak [8]. Similarly, we hypothesize that internet search data may indicate an increased incidence of peanut aspiration in children. Therefore, the purpose of this study is to utilize Google Trends searches relating to peanut aspiration to determine if more children aspirate peanuts after the 2017 guideline change.

This study was presented as a poster presentation at the Combined Otolaryngology Spring Meeting 2023 in Boston, MA under ASPO.

## Materials and methods

Phoenix Children’s Hospital Institutional Review Board exempted this study as no patient data were used. This study was conducted between October 1, 2022, and October 30th, 2022. Ten keyword combinations related to peanut aspiration were chosen by the principal investigator to try to account for possible search requests attempted by the public (choke on peanut, peanut + cough, peanut + choke, peanut + foreign body, peanut + aspirated, peanut + windpipe, peanut + bronchoscopy, peanut + inhaled, peanut + airway, peanut + lung). These keywords were then inputted into the Google Trends homepage using “United States,” “all categories,” and “web search” filters.

Google Trends

Google Trends measures the popularity of a keyword term searched in a given week compared to the popularity of all search terms in that week. The data are presented as relative search volume (RSV), yielding a value between 0 and 100, with 0 being of very little interest and 100 being the highest popularity [9,10]. We compiled US peanut aspiration-related search data by searching the 10 key terms listed above on the Google Trends platform accessed at https://trend.google.com for each year from 2012 to 2021. Then, the mean and median RSVs were calculated to compare the relative popularity of these searches divided into two periods: before the 2017 change in guidelines (from January 1, 2012, to December 31, 2016) to after the guideline change (January 1, 2017, to December 31, 2021).

Statistical analysis

Statistical analyses were conducted with SPSS Version 28 (IBM Corp., Armonk, NY, USA). The median percent change for each search term was calculated. The Mann-Whitney U test was used to compare the median RSVs. The data were analyzed at a 95% confidence level, and p-values less than 0.05 were determined to be statistically significant.

## Results

**Figure 1:**
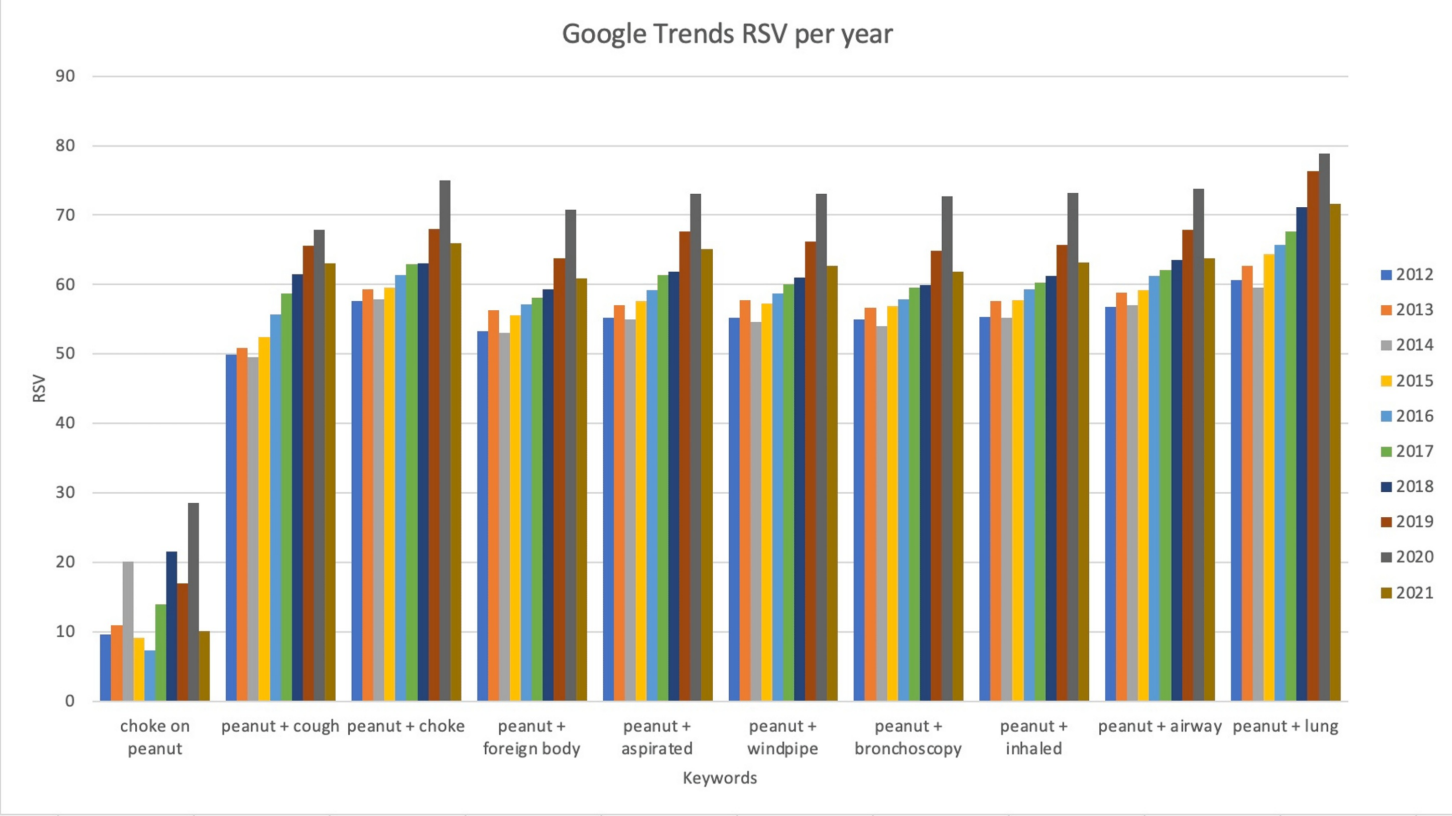
Annual RSV values for each of the 10 keywords from 2012 to 2021. RSV = relative search volume

The annual changes in search data for all 10 keywords from 2012 to 2021 are presented in Figure 1. The highest RSV occurred among all keywords during the year 2020 (Figure 1). In 2020, the combination “peanut + lung” was found to have the highest RSV of 79, followed by “peanut + airway,” “peanut + inhaled,” “peanut + windpipe,” and “peanut + aspirated” at an RSV of 73 (Figure 1). There was a decreasing trend in 2021; however, the RSV for the majority of keywords searched still remained higher than RSVs before 2017 (Figure 1).

There was an overall statistically significant increase in RSV for all 10 queried search terms when comparing the five years before and following January 2017. When pooling all terms, the median RSV increase was 13.8% (p < 0.001). Furthermore, “choke on peanut” and the combination of “peanut and cough” had the highest median RSV increases from 9.6 to 17, a change of 60%, and 50.8 to 63.1, a change of 23% (see Table 1).

**Table 1:** Median RSV values for peanut aspiration-related search terms pre- and post-AAP guideline changes. All significant at p < 0.05. AAP = American Academy of Pediatrics; RSV = relative search volume

Keyword search	2012–2016 median RSV	2017–2021 median RSV	% Change
Choke on peanut	9.58	17.0	60%
Peanut + Cough	50.8	63.1	23%
Peanut + Choke	59.3	66.0	13%
Peanut + Foreign body	55.6	60.8	14%
Peanut + Aspirated	57.1	65.1	16%
Peanut + Windpipe	57.3	62.7	14%
Peanut + Bronchoscopy	56.7	61.8	14%
Peanut + Inhaled	57.6	63.2	13%
Peanut + Airway	58.8	63.8	13%
Peanut + Lung	62.7	71.6	17%

## Discussion

The scientific literature has seen a growing interest in investigating Google Trends as a tool for monitoring disease epidemiology, public health awareness campaigns, and shifts in societal interest in certain healthcare topics [7-11]. Given that Google is the most widely used internet search engine by the public and health-related searches currently account for 4.5% of all total searches, Google Trends could be a valuable resource for gaining insights on health-related behaviors and disease patterns [11,12].

For example, previous studies have attempted to investigate Google Trends as a method to monitor the effects of major societal policy changes. A study by Ghomeshi et al identified a significant increase in search-related inquiries on Google regarding permanent forms of contraception after the overturn of *Roe vs. Wade* [13]. Regarding examining disease association relationships, a study by Tse et al. found correlations regarding the seasonal incidences of bronchiolitis and croup between hospital data and higher Google Trend searches [11]. Similarly, a study by Wang et al. found that Google search terms related to salmonellosis such as “hypovolemic shock,” “poor sanitation,” “hotel,” and other terms related to foods associated with *Salmonella* could correlate with the infectious disease outbreaks [14].

In our study, we found that since the change in the AAP guidelines in 2017, there has been a statistically significant increase in online search terms for peanut aspiration. The median RSV for all terms pooled together was 13.6% (p <0.001) after the change in AAP guidelines. This increase may be reflective of increased aspiration events, or at least increased concern for aspiration. These results are consistent with a retrospective cohort study by Leung et al. that discovered that more children in Australia were being admitted for peanut aspiration after the publication of the LEAP study in 2015 [3]. Although this study is by a single institution and contains a low sample size, it supports the notion derived from our findings [3].

The results of this study support the need to emphasize preventive efforts to avoid foreign body aspiration in children. If families want to introduce peanuts to their children, they should be closely counseled on the safe introduction of hard foods such as peanuts, or rather utilize infant-safe peanut products such as powder, dissolvable puffs, butter, or paste to prevent dangerous aspiration events [3,15]. Families should also be aware of signs of aspiration, as 15% of cases may go unnoticed [16]. Choking is present in 75% to 90% of cases; however, children can also present with persistent cough and difficulty breathing which could be misconstrued as illness or asthma [1]. Not only can peanut aspiration lead to potentially deadly airway obstruction, but it can also lead to infections such as pneumonia, abscesses, and bronchiectasis [3].

Several limitations should be noted. Google Trends can be used to gain public interest in healthcare topics but does not provide data from other search engines such as Bing, Yahoo, etc., or those without internet access. RSV changes can be related to other current events, decreasing the reliability of attributing them directly to AAP guidelines. Additionally, an increase in the RSV values could also be a result of the growing use of the internet for health-related topics over time. As Large Language Models (LLMs) such as ChatGPT become increasingly popular among parents, there is a potential for these tools to be widely used in seeking information related to their children’s health, including topics such as peanut consumption and choking hazards. Future research could explore how parents utilize LLMs in making decisions about introducing peanuts to their children and managing potential choking risks. This could help in understanding the impact of LLMs on health information-seeking behavior and its influence on real-world health outcomes [17,18]. Google Trends can also be viewed as an indirect measure of public interest rather than utilizing direct hospital admission data to monitor the change in peanut aspiration. However, institutional case series likely lack sufficient cases for meaningful comparison, and national databases coding for aspiration typically do not specify the nature of an aspirated foreign body. Search data analysis thus represents the most feasible approach to this research question.

## Conclusions

The change in AAP guidelines correlates with a rise in online searches regarding peanut aspiration. This result may be reflective of increased aspiration events, or at least a heightened concern for aspiration. The present findings emphasize the importance of advising families on infant-safe peanut products to reduce the risk of potentially dangerous aspiration events.
